# Additional phase information from UV damage of selenomethionine labelled proteins

**DOI:** 10.1107/S0909049511004092

**Published:** 2011-03-11

**Authors:** Daniele de Sanctis, Paul A. Tucker, Santosh Panjikar

**Affiliations:** a ESRF, Structural Biology Group, 6 rue Jules Horowitz, 38043 Grenoble Cedex, France; b EMBL Hamburg Outstation, c/o DESY, Notkestrasse 85, D-22603 Hamburg, Germany

**Keywords:** UV-RIPAS, SeMet, experimental phasing, radiation damage

## Abstract

Currently, selenium is the most widely used phasing vehicle for experimental phasing, either by single anomalous scattering or multiple-wavelength anomalous dispersion (MAD) procedures. The use of the single isomorphous replacement anomalous scattering (SIRAS) phasing procedure with selenomethionine containing proteins is not so commonly used, as it requires isomorphous native data. Here it is demonstrated that isomorphous differences can be measured from intensity changes measured from a selenium labelled protein crystal before and after UV exposure. These can be coupled with the anomalous signal from the dataset collected at the selenium absorption edge to obtain SIRAS phases in a UV-RIPAS phasing experiment. The phasing procedure for two selenomethionine proteins, the feruloyl esterase module of xylanase 10B from *Clostridium thermocellum* and the *Mycobacterium tuberculosis* chorismate synthase, have been investigated using datasets collected near the absorption edge of selenium before and after UV radiation. The utility of UV radiation in measuring radiation damage data for isomorphous differences is highlighted and it is shown that, after such measurements, the UV-RIPAS procedure yields comparable phase sets with those obtained from the conventional MAD procedure. The results presented are encouraging for the development of alternative phasing approaches for selenomethionine proteins in difficult cases.

## Abstract 2

Successful examples of ultraviolet radiation-damage-induced phasing with anomalous scattering from selenomethionine protein crystals have been demonstrated.

## 1. Introduction

Over the past 20 years the use of anomalous scattering information has become a routine means to determine a protein crystal structure (Hendrickson, 1991 ▶). In particular, selenium has been the most widely used element owing to its easy incorporation in labelled methionine, which is relatively abundant in protein sequences. It is an extensively used heavy-atom derivative (Hendrickson *et al.*, 1990 ▶), and the necessity for trial-and-error heavy-atom soaks has decreased over the years. As a consequence the use of anomalous dispersion techniques is increasing, and gradually replacing the more traditional isomorphous replacement techniques (single or multiple isomorphic replacement), in which intensity differences between heavy-atom derivatized crystals and native ones are used to calculate experimental phases. Very recently this phasing protocol has been re-applied in radiation-damage-induced phasing (RIP), where the difference in intensities induced by radiation damage was used as a phasing tool (Ravelli *et al.*, 2003 ▶). An extension of this method involving the use of the difference between an anomalous diffraction dataset with a radiation damaged one is termed radiation-damage-induced phasing with anomalous scattering (RIPAS) (Zwart *et al.*, 2004 ▶). Successful applications of these techniques have been achieved with the site-specific effects on sulfurs in disulfide bridges (Ravelli *et al.*, 2003 ▶; Banumathi *et al.*, 2004 ▶; Weiss *et al.*, 2004 ▶), triiodides (Evans *et al.*, 2003 ▶), brominated uridine (Ravelli *et al.*, 2003 ▶; Schiltz *et al.*, 2004 ▶) and mercury derivatives (Ramagopal *et al.*, 2005 ▶). Limitations of these phasing protocols are mainly due to the deleterious effect that a high X-ray dose has on a protein crystal. X-ray radiation damage induces many changes to the protein structure and to the solvent, resulting in a consistent number of damaged sites and in a decrease of the diffraction quality of the crystal. Recently, as an alternative to X-rays, UV radiation has been used to induce specific changes in the macromolecule, which only marginally affects the quality of the diffraction (Nanao & Ravelli, 2006 ▶), while inducing more specific changes to the protein structure. This method was named UV-RIP for ultraviolet radiation-damage-induced phasing.

The most striking effect of UV radiation damage to protein crystals, as for X-ray radiation, is the breakage of disulfide bonds, and this technique has been extended also to a non-disulphide-containing protein (photoactive yellow protein), which contains a chromophore, *p*-coumaric acid, covalently bound through a thioester linkage to a cysteine. Upon UV irradiation, the sulfur–carbon bond is disrupted (Nanao & Ravelli, 2006 ▶).

In a very recent study we have shown that crystals of selenomethionine (Mse) proteins can be damaged when exposed to UV (Panjikar *et al.*, 2011 ▶). The damage was very specific and mainly localized on the Se atoms. The differences in intensities recorded before and after exposing the crystals to UV radiation from a 266 nm laser (an energy far below the absorption edge of selenium) were sufficient to locate the Se atom substructure and to phase the protein structure by the UV-RIP technique.

Here we use the UV damage to Mse protein crystals to demonstrate the possibility of UV-RIPAS phasing and compare its efficacy with two-wavelength MAD phasing. Three datasets were collected. The first dataset was collected at the absorption edge (pk) of selenium, the second at the inflection point (ip) and the third at an energy far below the absorption edge after 50 min exposure to a 266 nm UV laser. UV-RIPAS experiments were performed with the first and last datasets, combined in a SIRAS phasing in the *SHELX* program suite and the results compared with the two-wavelength MAD method (using the first and second datasets). Evidence for phases of comparable quality is shown for two examples and the potential applications in other phasing protocols are discussed.

## 2. Experimental procedures

### 2.1. Target structures and experimental set-up

Two different Mse proteins were used in this study, the feruloyl esterase (FAE) module of xylanase 10B from *Clostridium thermocellum* (PDB code 1GKK) and the chorismate synthase (CHSYNT) from *Mycobacterium tuberculosis* (PDB code 2O11). FAE is composed of 297 residues and crystallizes in the *P*2_1_2_1_2_1_ space group, with two molecules in the asymmetric unit and a solvent content of 58%. It contains eight Se and four Cd atoms per monomer. Purification and crystallization protocols have been reported earlier (Prates *et al.*, 2001 ▶). The FAE crystal size used in this experiment was about 200 × 50 × 40 µm. CHSYNT contains 407 residues and crystallizes in space group *P*6_4_22, with one molecule in the asymmetric unit and a solvent content of 73% (Bruning *et al.*, 2011 ▶). The protein contains 11 selenomethionines. The CHSYNT crystal size was about 80 × 80 × 50 µm.

Diffraction data were collected at the ESRF beamline ID23EH1 (Nurizzo *et al.*, 2006 ▶). The X-ray beam was focused to a size of 40 × 30 µm at the sample position. A 266 nm laser (Teem photonic, SNU-02p) has been installed at the beamline and the arrangement is as used by Vernede and colleagues [for reference, see Fig. 1(*b*) from Vernede *et al.* (2006 ▶)]. The average power of the laser source is 5 mW, for a repetition rate of 7 kHz and a pulse width of 400 ps. The resulting UV spot at the sample position is much larger than the X-ray beam, and has a measured power of 1.4 mW, corresponding to about 10^15^ photons s^−1^ over a 880 × 670 µm area, giving a flux density of 1.7 × 10^15^ photons s^−1^ mm^−2^.

### 2.2. Data strategy and collection

The data collection strategy for all datasets was calculated using *BEST* (Bourenkov & Popov, 2010 ▶), as implemented in the *DNA* software pipeline. We applied sensible modifications to the collection plan to keep the total absorbed dose well below one-third of the maximum recommended dose of 30 MGy (Owen *et al.*, 2006 ▶) for all datasets collected from the crystals. The dose was calculated using the program *RADDOSE* (Paithankar & Garman, 2010 ▶). For each of the crystals a first dataset was collected at the peak of the absorption energy (‘pk’ dataset), a second at the inflection point (‘ip’ dataset) and a third dataset was collected at a low-energy remote of 12 keV (‘after’ dataset) on a ‘fresh’ part of the crystal following a 50 min exposure to UV. *CHOOCH* (Evans & Pettifer, 2001 ▶) was used to evaluate the energies at which the pk dataset and ip dataset were collected. Using *RADDOSE*, the total absorbed X-ray dose for the pk and ip datasets was calculated to be 2.43 and 0.62 MGy, and, for the low-energy remote, 1.99 and 0.49 MGy, for FAE and CHSYNT, respectively.

The crystals were exposed to the laser-derived UV radiation for 50 min, during which time they were oscillated once around the same rotation range (25 min) used for collecting the X-ray data, and then round the equivalent rotation range 180° away (25 min), with the objective of maximizing the damage. It should be noted that the UV and X-ray beam were co-axial.

We chose this UV exposure plan in order to address the limited penetration depth, reported in the literature (Nanao & Ravelli, 2006 ▶), of UV into a protein crystal and to damage the maximum volume of the crystal exposed in the ‘after’ data collection. Recently we showed that the expected UV penetration depth in Mse protein crystals is around 40 µm for FAE and 100 µm for CHSYNT (Panjikar *et al.*, 2011 ▶), which is more than enough to damage the bulk of the two different crystals used in this study.

### 2.3. Data processing

All data were indexed and integrated using *XDS* (Kabsch, 2010 ▶) and scaled using *XSCALE* (Kabsch, 2010 ▶). Scaled dataset files were converted to *SCALEPACK* format with the software tool *XDS2SCA* (Ravelli, unpublished). *SHELXC* (Sheldrick, 2010 ▶) was used to prepare the input files for *SHELXD* (Schneider & Sheldrick, 2002 ▶) and to analyze the anomalous and the isomorphous signal of the collected data. The resolution for UV-RIPAS phasing was chosen such that 〈Δ*F*/σ(Δ*F*)〉 was greater that 1.5. *F*_A_ values were calculated using the MAD and the SIRAS options in *SHELXC*. *SHELXD* was used to locate the substructure using the two-wavelength MAD and the SIRAS protocols. In the SIRAS protocol the ‘after’ dataset, which was collected at an energy below the absorption peak, was used as native, and the ‘pk’ dataset as the anomalous derivative. In both cases, 100 *SHELXD* trials in Patterson seeding mode were performed. We used a beta-version of *SHELXE* (Sheldrick, 2010 ▶) to calculate initial phases and improved phases, after density modification was carried out using the sphere of influence method. This newest version of *SHELXE* includes an autotracing feature (three cycles of autotracing alternating with new phase calculation and density modification) which was used to calculate initial phases and perform 100 cycles of density modification. Initial phases, prior to density modification, were obtained using *SHELXE* with no density modification cycles (-m0 flag).

## 3. Results

### 3.1. MAD FAE

FAE contains a very strong anomalous signal, which was provided by the eight Se and four Cd atoms per molecule. Data collection statistics are shown in Table 1 ▶.

**Figure 1:**
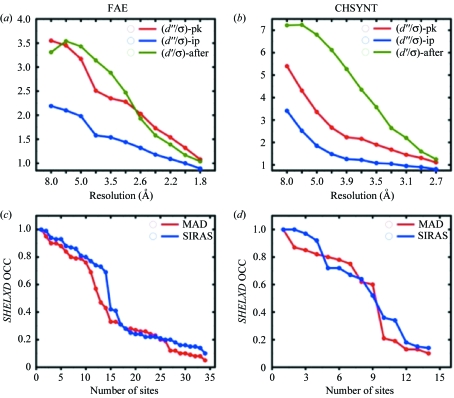
(*a*) Plot of *SHELXC* output, showing 〈*d*′′/σ〉 for the peak (in red) and inflection point (in blue) datasets of FAE. Usable anomalous signal is up to 1.79 Å resolution. The 〈*d*′/σ〉 isomorphous signal in the ‘after’ dataset is shown in green. (*b*) Plot of *SHELXC* output, showing 〈*d*′′/σ〉 for the peak (in red) and inflection point (in blue) datasets of CHSYNT. Usable anomalous signal is up to 2.7 Å resolution. The 〈*d*′/σ〉 isomorphous signal in the ‘after’ dataset is shown in green. (*c*) Plot of the substructure atom occupancy for FAE. After the analysis we noticed that the MAD substructure presents some of the Mse in double conformations and that the located Cd atoms have lower occupancy. Hence the drop in occupancy is not as clear as in the case of SIRAS. (*d*) Atom occupancy for CHSYNT. In this case SIRAS does not present a clear drop, owing to the presence of extra damaged sites (see text)

Analysis with *SHELXC* shows that a strong anomalous signal in the ‘pk’ and ‘ip’ datasets is present up to the maximum resolution of the data (1.79 Å) with 〈*d*′′/σ〉 of 1.08 and 33.6% anomalous correlation. The statistics produced by *SHELXC* were used to compare UV-RIPAS datasets with MAD (Fig. 1 ▶) datasets. With such a good anomalous signal, substructure solution using the MAD experiment was of course straightforward. *SHELXD* was able to find all 16 Se and 8 Cd atoms present in the asymmetric unit, resulting in a very good correlation coefficient (CC) between observed and calculated *E* values (CC_all_/CC_weak_ = 50.55/34.90).

*SHELXE* was used first to calculate phases from the substructure giving a mean figure of merit (FOM) of 0.368, without performing any cycles of density modification and then with three cycles of autotracing. These were alternated with 100 cycles of density modification, and achieved the building of 557/564 residues, with a resulting mean FOM of 0.761. The correlation of the calculated map with the final deposited model is 85% (Table 2 ▶).

### 3.2. UV-RIPAS FAE

Analysis of the anomalous and isomorphous signals of the collected data showed a surprisingly large isomorphous component owing to the presence of specific UV damage in the protein structure, as proven by the control experiment shown below (Fig. 1*b* ▶, Table 5). This isomorphous signal, together with the existing anomalous signal from the dataset collected at the peak, was used to perform an UV-RIPAS experiment. The ‘after’ dataset still showed some anomalous signal, perhaps owing to the Cd atoms and the partial damage to the Se atoms.

It is intriguing to notice how strong the isomorphous signal is at low resolution even compared with the anomalous one, as shown in Fig. 1(*b*) ▶. Contrary to what has been reported in other cases of UV-RIP phasing (Nanao *et al.*, 2005 ▶), no downscaling of the ‘after’ dataset was necessary with *SHELXC*, as in both examined cases the substructure was easily determined.

As observed in other cases of UV-RIP phasing (Nanao *et al.*, 2005 ▶; Panjikar *et al.*, 2011 ▶), these UV-RIPAS experiments also show the absolute value of CC_all_/CC_weak_ to be lower than that obtained with a MAD experiment but nevertheless they still clearly indicate a good solution. The CC_all_/CC_weak_ values of the solution were 22.52/15.84, and, although very low, they clearly discriminate between ‘correct’ and other ‘wrong’ solutions. The substructure determined by the SIRAS protocol as implemented in *SHELX* matched that determined by MAD except for the Cd sites, as only one Cd site was found, which was close to a selenomethionine residue. The substructure was then fed into *SHELXE*, which was able to phase the structure, although initial phases were clearly much poorer than those given by the MAD experiment (mean FOM of 0.155, pseudo-free CC of 16.00%, phase error of 81.1°). This is probably due to the fact that the substructure determined by MAD includes the sites corresponding to the Cd atoms. Using the usual density modification protocol in combination with autotracing resulted in a mean FOM of 0.724, a pseudo-free CC of 76.50% and a mean phase error of 43.6°. The refined and the new sites found were recycled for calculation of new phases and the phasing process was repeated. New phasing and density modification resulted in a slight improvement in the quality of the map, with a mean FOM of 0.730, pseudo-free CC of 77.10% and a phase error of 43.1°. A total of 551 residues were built into the electron density. The correlation coefficient of the calculated map with the final model was 86% (Table 2 ▶).

### 3.3. MAD CHSYNT

CHSYNT data collection statistics are reported in Table 1 ▶. The molecule contains 11 Se atoms in the asymmetric unit.

MAD datasets were collected at the peak and inflection point energies. Prior to substructure solution, data were prepared with *SHELXC*, and gave good statistics for the anomalous scattering. A very strong signal was present in all resolution ranges and analysis of 〈*d*′′/σ〉 and of the correlation between the two datasets, up to 3.0 Å resolution, gave 1.31 and 33.6, respectively.

*SHELXD* was able to clearly find nine selenium positions out of 11 (the other two seleomethionines are disordered), with excellent correlation coefficient (CC_all_/CC_weak_) values of 49.88/33.58. The *SHELXE* phasing experiment resulted in a mean FOM of 0.224 and a pseudo-free CC of 20.21% (phase error 77.1°). *SHELXE* density modification with autotracing resulted in a final mean FOM of 0.756 and a pseudo-free CC of 79.81% (phase error 40.9°). The last cycle of autotracing succeeded in placing 331 residues out of 407 in the electron density.

### 3.4. UV-RIPAS CHSYNT

Similar to the FAE experiment, the crystal was exposed to the UV light source for a total time of 50 min, as described in §2. The ‘after’ dataset was then used in combination with the peak dataset to perform a SIRAS experiment. Analysis with *SHELXC* shows that a consistent isomorphous signal was present when comparing the two datasets, as shown in Fig. 1(*b*) ▶. *SHELXD* managed to locate at least nine atoms, with CC_all_/CC_weak_ values of 28.28/19.76, although the drop in atom occupancy was not as sharp as that seen in the MAD experiment analysis (Fig. 1*d* ▶). Initial phases led to an FOM of 0.276, a pseudo-free CC of 21.31% with a phase error of 73.9°. *SHELXE* was run with 100 cycles of density modification. Autotracing placed 341 residues in the electron density, leading to a final FOM of 0.738 and pseudo-free CC of 78.37% for a phase error of 42.4°. Recycling of the updated substructure in *SHELXE* slightly improves the starting phases resulting in a mean FOM of 0.311, a pseudo-free CC of 26.08% and a phase error of 71.9°. Final density modification also leads to improved results, with a mean FOM of 0.740 and a pseudo-free CC of 78.72% with a phase error of 42.3° (Table 2 ▶).

## 4. Discussion

### 4.1. MAD *versus* UV-RIPAS

We have presented here the results of using UV radiation damage to selenomethionine in combination with the anomalous signal to solve the structure of two proteins, the FAE module of xylanase 10B from *Clostridium thermocellum* (PDB code 1GKK) and CHSYNT from *Mycobacterium tuberculosis* (PDB code 2O11). Classical two-wavelength MAD and UV-RIPAS, treated as a SIRAS experiment, with the ‘after’ dataset as native and the ‘peak’ as derivative, were used to solve both structures. *SHELXD* was used to find the positions of the Se atoms and *SHELXE* to calculate experimental phases and then to improve them by density modification cycles, interspersed with poly-ala chain tracing.

In the case of FAE, the two phasing protocols led to comparable results. The selenium sites found were the same in the two substructure determination protocols, but only one Cd atom was found in the SIRAS procedure. This Cd atom interacts with the Se atom of Mse889, while the other three Cd atoms were not affected by UV radiation and therefore were not located as SIRAS sites. This implies that electrostatically coordinated Cd atoms that were not within the vicinity of UV absorbing residues were not significantly damaged by UV light.

In the case of CHSYNT, all possible selenium sites were found with either of the two protocols for the substructure solution. It is worth noting that the absolute values of CC_all_ and CC_weak_ in *SHELXD* were higher in the MAD than in the UV-RIPAS case. The comparison between the two methods in terms of substructure solution, initial phases, and final density and phases is reported in Table 2 ▶. The UV-induced SIRAS experiment on the selenomethionine derivative protein crystal was a straightforward phasing experiment, which provided phases of comparable quality to that of the MAD analysis even prior to any density modification cycles. The quality of the final map resulting from UV-RIPAS was indistinguishable from the MAD one, as shown by the autotracing results.

Difference Fourier map peaks of the substructures for the two experiments were compared (Table 3 ▶). The σ level of the map calculated with *SHELXE* (using *F*_A_ and α) is shown along with the occupancy of the substructure as determined by *SHELXD*. From the structure analysis we noticed that, while the most intense peaks calculated with MAD phasing corresponded to more buried methionines with lower *B*-factors, the sites found from the SIRAS synthesis (and the electron density of the substructure) ranked in a different order. This demonstrated that the difference between the two cases is the sensitivity to UV of the Se atoms which was not equivalent for all sites. It was therefore evident that the substructures determined *via* the two procedures can be complementary.

In addition to the selenium sites, new peaks were identified in the SIRAS case in the side-chains of Asp980 (6.6σ), Cys967 (6.2σ) Leu977 (6.0σ) and on the main chain of Ala1012 (6.4σ), indicating a loss of electron density on these residues. This was most likely due to a structural rearrangement in consequence of the damage to Se atoms. Relevant negative peaks were found near selenomethionine residues Mse863 and Mse1031, and were evidently due to conformational changes induced by UV irradiation.

This UV-RIPAS-induced experiment could benefit from a larger substructure compared with the MAD experiment, or in combination with MAD. UV is known to induce other differences in the crystal, and additional sites were located in the refinement of the sites performed by *SHELXE* in the last cycle of phase calculation. We showed how evident improvement can be obtained by recalculating experimental phases with the new array of sites in this case.

A very similar scenario to that which we observed for FAE was seen for CHSYNT. The peak height in units of map r.m.s. for the substructure density calculated with *SHELXE* is shown in Table 4 ▶. The numbering of the residues was kept consistent with the deposited PDB entry. In this case it is clear how UV radiation can play an important role in enhancing the isomorphous signal of Se atoms. Additional loss of electron density was identified on the carboxyl group of Glu134 and Asp373 (12.8σ and 9.6σ, respectively), which are found in proximity to the selenomethionine Mse89 side-chain. Other damage/sites were near Ile63 (8.2σ) on the acetate ion ACT408 (7.8σ) and on the carboxyl group of Asp185 (7.1σ) and Glu9 (7.0σ). Other sites with lower σ levels were also found. It is clear that including these sites and re-running the phasing procedure can provide improved initial phases.

Also for CHSYNT it is intriguing to note that the sites of maximum damage occur in a different order if we compare the MAD dataset with the SIRAS one. In other words, the Se atoms are not contributing in the same way if we consider their anomalous signal or the combination of anomalous with the isomorphous signal caused by UV irradiation.

### 4.2. Control experiment

In order to determine whether the isomorphous difference that we observed during the UV-RIPAS phasing was due to the dispersive signal of Se only or the UV damage to the Se atoms, we performed a control experiment on only an FAE crystal, collecting a ‘pk’ dataset followed by a low-energy remote at 12 keV. This was compared with a ‘pk’ dataset followed by a low-energy remote dataset collected after 50 min UV exposure. All data were collected from the same large crystal (of size ∼300 × 200 × 50 µm). The data were analysed using *SHELXC* to prepare for SIRAS phasing. The output from *SHELXC* is shown in Table 5 ▶ and clearly indicates that the dispersive signal, although detectable, has a very limited effect if compared with the UV exposed signal. Substructure determination with *SHELXD*, using the same procedure described in §2, was not successful in the first case (‘pk’ and ‘low-energy remote before UV exposed’) while it was easily solved in the second case (‘pk’ and ‘low-energy remote after UV exposure’).

### 4.3. Future perspective

During these experiments, various phasing procedures were tried. We were able to phase the protein structure *via* RIP phasing, with data collected at low-energy remote away from the absorption edge (Panjikar *et al.*, 2011 ▶). This is the only successful case of RIP with Mse proteins of which we are aware. While this article was being prepared, alternative phasing protocols were investigated. A SIRAS phasing protocol was successfully tried in other scenarios, such as collecting two datasets at the peak energy with a UV exposure in between. It is also intriguing to note that the crystals exposed to UV still retain sufficient anomalous signal from Se atoms to allow the substructure determination, and that no major change is observed in the X-ray energy absorption spectra. Whether this is a consequence of the limited penetration of UV inside the crystalline material (which may be overestimated in our calculations) is currently under investigation. In any case, the UV-damaged Mse dataset can always be used as a highly isomorphous artificial ‘native’, which can then be combined with traditional anomalous dispersion datasets. We showed that the substructure of the damaged sites can be determined independently from the anomalous data, hence the substructure determination and experimental phasing are independent of those calculated *via* anomalous dispersion. We can imagine that the calculated phase distribution from the two techniques could be combined for more accurate phase estimates.

As anticipated, the mechanism behind UV damage to seleniomethionine is still unclear. Panjikar *et al.* (2011 ▶) showed that Mse residues absorb UV radiation within the wavelength range 240–270 nm and speculated that these direct effects induce the damage to Se atoms. Determination of the ration­ale behind the sensitivity of Mse and whether the local or global environment of the residue plays a role requires analysis of more UV-damaged Mse proteins in combination with complementary, in particular spectroscopic, techniques.

## 5. Conclusions

Selenium labelling of methionine is nowadays probably the most common way to obtain experimental phases in protein crystallography. In the present work we demonstrated how the combination of anomalous scattering from Se atoms and the isomorphous differences induced by UV radiation damage on the same atom is a powerful technique for calculating initial experimental phases. The combination of anomalous and isomorphous signals to perform a UV-RIPAS experiment leads to initial phases comparable in quality to those obtained by a conventional MAD experiment. We showed how the intensities of the peaks in the substructure density (hence the site occupancy) obtained from MAD differ from those resulting from the UV-RIPAS protocol. This suggests that the sites arising from UV damage can have a different contribution to phasing than the same sites determined by MAD.

Analysis of the isomorphous signal as a function of resolution for both cases investigated here indicates a strong signal which can be even higher than the anomalous signal at low resolution. We believe that in some difficult phasing experiments with Se atoms this additional information can be used to determine the substructure, as well as giving enhanced phase information. In particular, it is noteworthy that the isomorphous difference is higher in general than the anomalous one. It is foreseeable that in the special case of a low-resolution diffracting crystal and a small substructure, with limited contribution to the phasing power from the anomalous and dispersive signals, isomorphous differences from UV damage could be the crucial technique for obtaining additional isomorphous signal for substructure solution and phasing.

One additional advantage of the UV-RIPAS phasing protocol compared with the MAD one is the amount of data needed. The ‘after’ dataset, treated as native, can have the Friedel pairs merged during data processing in order to achieve the required completeness. This can be particularly useful for cases which crystallize in low-symmetry space groups and for highly radiation sensitive protein crystals.

## Figures and Tables

**Table 1:** Data collection statistics for FAE, CHSYNT and the FAE control Where two numbers are given, the second number refers to the highest-resolution shell.

	FAE			CHSYNT			FAE control		
	pk	ip	after	pk	ip	after	pk	low-energy	after
Wavelength (Å)	0.979250	0.979450	1.0332	0.979250	0.979450	1.0332	0.979250	1.0332	1.0332
Total frames per dataset	100	100	100	100	100	110	100	100	100
Space group	*P*2_1_2_1_2_1_	*P*2_1_2_1_2_1_	*P*2_1_2_1_2_1_	*P*6_4_22	*P*6_4_22	*P*6_4_22	*P*2_1_2_1_2_1_	*P*2_1_2_1_2_1_	*P*2_1_2_1_2_1_
Unit cell (Å)	*a* = 65.29	*a* = 65.30	*a* = 65.61	*a* = 131.89	*a* = 132.18	*a* = 131.93	*a* = 64.88	*a* = 65.05	*a* = 65.04
	*b* = 108.26	*b* = 108.28	*b* = 108.46	*b* = 131.89	*b* = 132.18	*b* = 131.93	*b* = 108.33	*b* = 108.64	*b* = 108.50
	*c* = 112.95	*c* = 112.99	*c* = 113.26	*c* = 159.79	*c* = 160.02	*c* = 160.19	*c* = 112.81	*c* = 113.14	*c* = 113.12
Resolution (Å)	1.78	1.78	1.79	2.30	2.70	2.50	1.90	2.00	1.90
Mosaicity (°)	0.127	0.127	0.117	0.076	0.123	0.106	0.088	0.135	0.104
Total reflections	287456/11841	287524/11823	322211/13239	438841/51740	273289/28181	344666/34808	305712/29727	260366/24521	278664/19885
Unique reflections	140862/8647	140953/8644	142288/9048	69081/8294	42960/4447	53967/5461	107327/12865	90311/10540	54444/5351
Redundancy	2.04/1.37	2.04/1.36	2.26/1.46	6.35/6.24	6.36/6.34	6.39/6.37	2.84/2.31	2.88/2.32	5.11/3.71
Completeness (%)	95.0/61.8	95.7/80.0	95.1/63.9	100.0/100.0	99.9/100.0	99.9/100.0	88.0/65.1	83.9/61.1	85.0/52.4
*I*/σ(*I*)	13.78/2.62	14.64/2.57	12.06/1.79	13.64/2.03	14.37/2.16	19.63/2.64	9.56/2.22	10.07/1.78	13.84/2.92
*R*_merge_ (%)	4.3/21.7	4.1/23.1	6.1/35.2	11.6/92.7	13.0/90.6	8.3/72.7	11.7/58.1	11.5/74.2	8.9/53.8
*R*_meas_ (%)	5.6/29.6	5.3/31.3	7.5/47.3	12.7/100.9	14.2/98.6	9.1/79.3	14.1/72.8	13.8/93.5	10.7/68.9
*R*_pim_ (%)	4.1/19.8	3.2/19.7	3.7/29.2	4.0/28.4	4.2/28.0	2.6/22.2	7.7/43.1	7.6/55.8	5.9/42.3
Anom-correl (%)	68/19	41/5	14/5	55/7	32/5	3/−1	36.4/2.4	0/−11	4.6/−5.6
Outer resolution shell	1.84–1.78	1.84–1.78	1.84–1.78	2.40–2.30	2.80–2.70	2.59–2.50	2.00–1.90	2.10–2.00	2.00/1.90

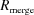
 = 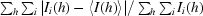, 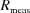 = 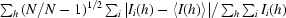, 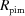 = 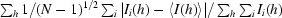.

**Table 2:** Substructure solution and phasing statistics for the different phasing protocols The numbers in parentheses refer to the highest-resolution shell. The last row contains the correlation coefficient of the final density modified map of *SHELXE* with the deposited structure.

	FAE MAD	FAE SIRAS	CHSYNT MAD	CHSYNT SIRAS
CC_all_/CC_weak_	50.55/34.90	22.52/15.84	49.88/33.58	28.28/19.76
Initial phase error (°)	68.4	81.1 (76.5)	77.1	73.9 (71.9)
Final phase error (°)	40.4	43.6 (43.1)	40.9	42.4 (42.3)
CC exp. map to final model	0.85	0.86	0.87	0.89

**Table 3:** Comparison of the FAE substructure peaks for the two phasing protocols Residue numbers are according to the deposited structure sequence of FAE (PDB code 1GKK). Substructure density peaks were calculated with *SHELXE* using *F*_A_ and α.

	FAE MAD		FAE SIRAS	
Residue number	Sigma level	*SHELXD* occupancy	Sigma level	*SHELXD* occupancy
Selenium				
A863	25.6	0.44	49.9	0.86
A889	56.1	0.95	36.8	0.88
A946	51.5	0.90	29.5	0.77
A955	19.6	0.34	13.5	0.42
A964	43.7	0.79	57.3	0.99
A975	61.9	1.00	40.7	0.93
A1024	49.9	0.79	36.2	0.74
A1031	52.5	0.69	37.4	0.80
B863	29.6	0.47	53.7	0.94
B889	56.8	0.88	37.5	0.88
B946	46.6	0.80	31.0	0.73
B955	18.8	0.34	16.7	0.41
B964	50.8	0.84	51.6	1.00
B975	54.9	0.90	39.9	0.93
B1024	44.8	0.76	39.4	0.69
B1031	32.0	0.56	40.1	0.81

Cadmium				
A3086	13.8	0.28	19.7	0.31
A3087	11.5	0.24	11.5	–
A3088	11.5	0.23	11.6	–
A3089	8.5	0.19	6.9	–
B3086	16.4	0.31	23.2	0.22
B3087	11.7	0.25	9.0	–
B3088	11.3	0.26	9.7	–
B3089	9.8	0.20	8.2	–

**Table 4:** Comparison of CHSYNT substructure peaks for the two phasing protocols Residue numbers are according to the deposited structure sequence of CHSYNT (PDB code 2O11). Substructure density peaks were calculated with *SHELXE* using *F*_A_ and α.

	CHSYNT MAD		CHSYNT SIRAS	
Residue number	Sigma level	*SHELXD* occupancy	Sigma level	*SHELXD* occupancy
Selenium				
A22	35.8	0.82	48.3	1.00
A89	43.8	1.00	54.5	1.00
A121	32.5	0.81	38.7	0.92
A205	33.3	0.81	22.1	0.52
A253	32.4	0.78	45.3	0.97
A281	25.4	0.60	34.0	0.64
A302	39.1	0.85	31.4	0.67
A314	33.8	0.75	29.4	0.72
A357	28.2	0.62	38.5	0.72

**Table:** (*a*) Analysis of the SIRAS with pk dataset and low-energy remote without UV exposure. SIRAS did not lead to substructure determination using the two datasets. (*b*) The corresponding statistics but using the same pk dataset and a low-energy remote collected after having exposed a second FAE crystal to UV for 50 min. It can be seen that the isomorphous signal (〈*d*′/σ〉) is strong. The complete substructure was determined using these two datasets with the SIRAS method, demonstrating the importance of specific UV damage. Correlation values of *E* from *SHELXD* were CC_all_ = 36.85, CC_weak_ = 26.57.

(*a*)											
Res.	8.0	6.0	5.0	4.0	3.5	3.0	2.6	2.4	2.2	2.0	1.80
*N*(data)	825	1103	1384	3152	3163	5695	8276	6457	9059	12517	11876
〈*I*/σ〉	28.3	25.6	24.6	26.2	23.3	17.8	11.6	8.6	6.7	4.3	2.0
% Complete	83.4	88.7	89.9	91.9	92.8	94.2	95.5	96.1	96.7	93.1	59.3
〈*d*′′/σ〉	1.70	1.72	1.63	1.35	1.34	1.27	1.17	1.10	1.03	0.94	0.82
〈*d*′/σ〉	2.70	2.42	2.28	2.36	2.20	1.78	1.39	1.23	1.14	1.01	0.88
*R*(isom)	0.094	0.095	0.098	0.091	0.101	0.113	0.145	0.184	0.221	0.285	0.351

**Table:** 

(*b*)											
Res.	8.0	6.0	5.0	4.0	3.5	3.0	2.6	2.4	2.2	2.0	1.80
*N*(data)	826	1103	1386	3150	3170	5692	8278	6469	9042	12526	11865
〈*I*/σ〉	28.3	25.6	24.6	26.2	23.3	17.8	11.6	8.6	6.7	4.3	2.0
% Complete	83.5	88.7	89.9	91.9	92.8	94.2	95.5	96.2	96.7	93.1	59.1
〈*d*′′/σ〉	1.70	1.72	1.63	1.35	1.34	1.27	1.18	1.09	1.04	0.94	0.82
〈*d*′/σ〉	4.39	5.09	4.83	4.52	4.09	3.45	2.39	1.95	1.61	1.29	0.97
*R*(isom)	0.135	0.200	0.202	0.177	0.186	0.209	0.224	0.250	0.260	0.299	0.354
